# Manic patients and sleep management: the role of polysomnography in clinical practice

**DOI:** 10.1192/j.eurpsy.2022.1036

**Published:** 2022-09-01

**Authors:** F. Pacchioni, M.C. Cavallini, L. Fregna, A. Sarzetto, F. Attanasio, B. Barbini, C. Colombo

**Affiliations:** 1 Università Vita-Salute San Raffaele, Psychiatry, Milano, Italy; 2 IRCCS San Raffaele Scientific Institute, Psychiatry - Mood Disorders, Milano, Italy

**Keywords:** Bipola Disorder, Polysomnography, mania, sleep

## Abstract

**Introduction:**

*Sleep plays a key role in the pathogenesis and clinic of mood disorders. However, few studies have investigated electroencephalographic sleep parameters during the manic phases of Bipolar Disorder (BD).*

**Objectives:**

Sleep management is a priority objective in the treatment of the manic phases of BD and the polysomnographic investigation can be a valid tool both in the diagnostic phase and in monitoring clinical progress.

**Methods:**

*Twenty-one patients affected by BD, manic phase, were subjected to sleep monitoring via PSG in the acute phase (at the entrance to the ward) and in the resolution phase (near discharge). All participants were also clinically* evaluated *using Young Manic Rating Scale* (*YMRS) Pittsburgh Sleep Quality Index (PSQI), Morningness-eveningness Questionnaire (MEQ) at different timepoints.*

**Results:**

*Over the hospitalization time frame there was an increase in quantity (Total Sleep Time) and an improvement in the quality and effectiveness of sleep (Sleep Efficiency). In addition, from the point of view of the EEG structure, clinical improvement was accompanied by an increase in the percentage of REM sleep.*

**Conclusions:**

*Sleep monitoring by PSG can be a valuable tool in the clinical setting both in the diagnostic phase, “objectively” ascertaining the amount of sleep, and in the prognostic phase, identifying electroencephalographic characteristics that can predict the patient’s progress and response to drug therapy. The improvement in effectiveness and continuity of sleep and the change in its structure that accompanies the resolution of manic symptoms also testifies how the regularization of the sleep-wake rhythm is to be considered a priority in treating manic phases.*

**Disclosure:**

No significant relationships.

